# Clinical experience with power-injectable PICCs in intensive care patients

**DOI:** 10.1186/cc11181

**Published:** 2012-02-04

**Authors:** Mauro Pittiruti, Alberto Brutti, Davide Celentano, Massimiliano Pomponi, Daniele G Biasucci, Maria Giuseppina Annetta, Giancarlo Scoppettuolo

**Affiliations:** 1 Department of Surgery, Catholic University Hospital, Largo F. Vito 1, 00168 Rome, Italy; 2 Department of Anesthesiology and Intensive Care, Catholic University Hospital, Largo F. Vito 1, 00168 Rome, Italy; 3 Department of Infectious Diseases, Catholic University Hospital, Largo F. Vito 1, 00168 Rome, Italy

## Abstract

**Introduction:**

In the ICU, peripherally inserted central catheters (PICCs) may be an alternative option to standard central venous catheters, particularly in patients with coagulation disorders or at high risk for infection. Some limits of PICCs (such as low flow rates) may be overcome with the use of power-injectable catheters.

**Methods:**

We retrospectively reviewed all of the power-injectable PICCs inserted in adult and pediatric patients in the ICU during a 12-month period, focusing on the rate of complications at insertion and during maintenance.

**Results:**

We collected 89 power-injectable PICCs (in adults and in children), both multiple and single lumen. All insertions were successful. There were no major complications at insertion and no episodes of catheter-related bloodstream infection. Non-infective complications during management were not clinically significant. There was one episode of symptomatic thrombosis during the stay in the ICU and one episode after transfer of a patient to a non-intensive ward.

**Conclusion:**

Power-injectable PICCs have many advantages in the ICU: they can be used as multipurpose central lines for any type of infusion including high-flow infusion, for hemodynamic monitoring, and for high-pressure injection of contrast media during radiological procedures. Their insertion is successful in 100% of cases and is not associated with significant risks, even in patients with coagulation disorders. Their maintenance is associated with an extremely low rate of infective and non-infective complications.

## Introduction

The use of peripherally inserted central catheters (PICCs) has many theoretical advantages in the ICU setting because these devices are associated with low-risk insertion, even in patients with altered coagulation and/or difficult neck anatomy [1]. PICC insertion can be carried out with no risk of pleura-pulmonary damage and with no clinically significant risk of local hemorrhage or hematoma, if compared with standard central venous catheters (CVCs) [2]. Furthermore, PICC insertion in the upper mid-arm is characterized by an easy dressing of the exit site, this benefit being particularly evident in patients with tracheostomy [3]. Although the issue is somehow controversial [4,5], PICCs are also usually considered a device at low risk for catheter-related bloodstream infection (CRBSI), which may be an additional advantage in acutely ill patients [2]. PICCs can also be used for central venous pressure monitoring [6], specifically when using polyurethane, open-ended catheters > 4 Fr, as long as they have no evidence of malfunction.

Although the potential benefit of PICCs in the acutely ill patient was proposed almost 10 years ago [7], some technical limitations have slowed their introduction into clinical practice in the ICU. The ICU patient usually requires high flow rates of intravenous infusion as well as simultaneous administration of potentially incompatible drugs, which should ideally be delivered through multiple-lumen catheters. Since standard PICCs are catheters with small caliber (typically, 4 to 5 Fr) and relevant length (30 to 40 cm on average), they are associated with a high resistance to flow. A single-lumen 4 Fr PICC may achieve a flow rate of 2 to 3 ml/minute (by gravity infusion) and 10 to 11 ml/minute (with pump); the flow rates of a single-lumen 5 Fr PICC are only slightly higher (3 to 4 ml/minute by gravity and 11 to 13 ml/minute with pump). Double-lumen PICCs have worse performance in terms of flow, because increasing the number of lumens reduces the lumen size and further decreases the flow rate.

The situation changed following the recent development of PICCs made of ultra-resistant polyurethane, which were originally introduced for use with the high-pressure pumps (so-called power injectors) commonly utilized for high-velocity infusion of contrast media during computed tomography scan and other radiological procedures. The rationale for these so-called power-injectable PICCs was the concern of potential mechanical damage to standard polyurethane and silicon PICCs when used during high-pressure injection. Power injectors may develop pressures as high as 300 psi (silicone catheters tolerate no more than 50 to 60 psi, and most polyurethane catheters approximately 100 psi). Several reports of mechanical damage to PICCs and other devices have been described in the literature, leading to an official warning from the US Food and Drug Administration [8] - which recommended that power injection should be done exclusively through venous access devices specifically registered for this use.

Such power-injectable PICCs - developed and marketed by several companies - have been shown to have the additional advantage of delivering infusions at a very high rate (3 to 5 ml/second = 180 to 300 ml/minute), if coupled with an appropriate infusion pump. Multiple-lumen PICCs were also developed shortly thereafter, and their use in the ICU started to spread. At present, several brands of power-injectable PICCs are available - single lumen (3, 4 or 5 Fr), double lumen (4 or 5 Fr) and triple lumen (6 Fr). They all share several features that make them particularly attractive for the ICU setting: low risk of mechanical and hemorrhagic complications at insertion, low risk of CRBSI, high flow (up to 300 ml/minute), easy monitoring of central venous pressure, low risk of lumen obstruction, and safe use for radio-diagnostic procedures. Most power-injectable PICCs also have a reverse-taper design at the proximal end; that is, the diameter of the catheter that enters the exit site is slightly thicker than the diameter of the rest of the catheter. This technical feature is potentially associated with reduced bleeding at the exit site soon after insertion, with increased stability of the PICC (reduced risk of dislodgement) and with protection against exit site contamination.

In the present paper, we report our preliminary experience with the use of power-injectable PICCs in adult and pediatric ICU patients.

## Materials and methods

We reviewed retrospectively all power-injectable PICCs inserted in acutely ill patients admitted to the shock/trauma ICU and the pediatric ICU of our institution (a 1,100-bed university hospital), during 12 months.

According to our hospital policies, indications for PICC insertion were (a) need for a central line for parenteral nutrition, and/or infusion of drugs that require a central line (pH < 5 or > 9, osmolarity > 500 mOsm/l, drugs associated with endothelial damage), and/or (b) central venous pressure monitoring, and/or (c) need for frequent blood sampling. Need for a central line with more than three lumens was considered a clear indication for a standard multiple-lumen CVC. Also, all PICCs were inserted as an elective procedure; all central lines inserted in emergency were standard CVCs. Overt sepsis was also considered a contraindication to PICC insertion. Other standard contraindications to PICC insertion, according to our hospital policies, were small deep veins of the arm (brachial/basilic vein < 4 mm), local contraindications due to specific arm conditions (skin infection, burns, orthopedic devices blocking the arm, previous axillary node resection due to breast cancer surgery, and so forth) as well as actual or impending chronic renal failure requiring an arteriovenous fistula. Severe arm edema and/or obesity are not considered contraindications to PICC insertion, although in these situations the brachial and basilic vein might be too deep ( > 3 cm) so the cephalic vein is preferentially cannulated.

**Figure 1:**
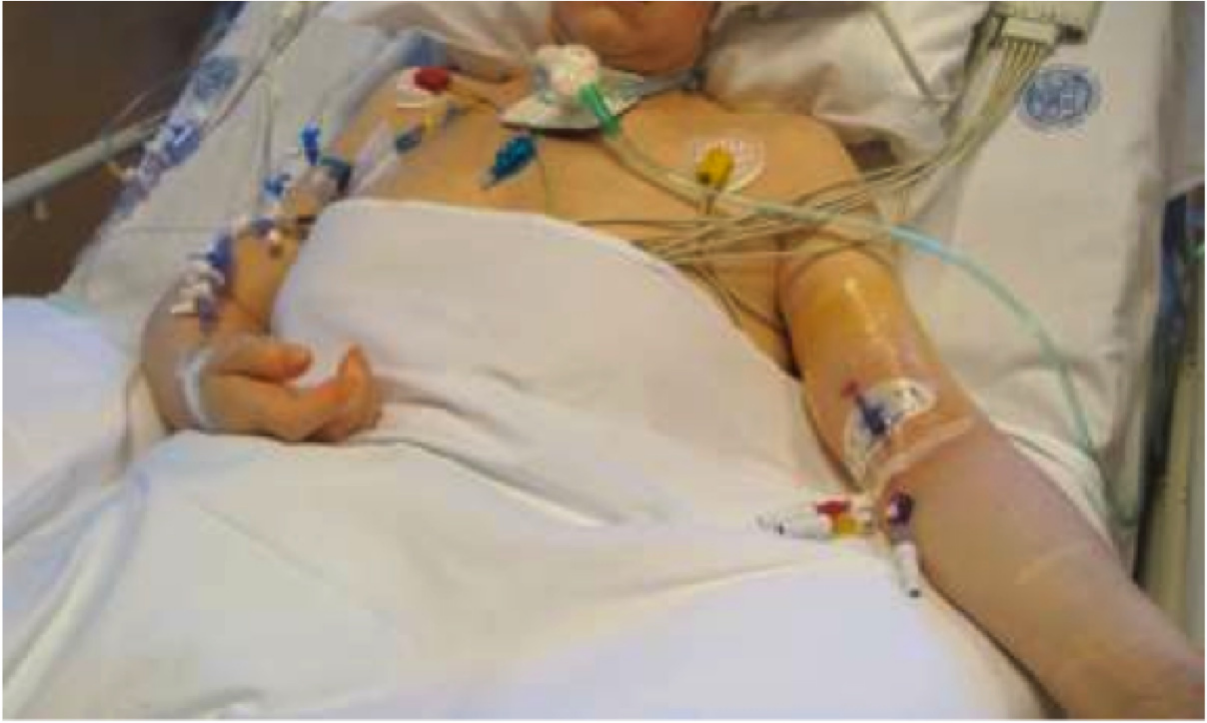
**Triple-lumen power-injectable peripherally inserted central catheter inserted in an adult patient in the ICU**.

**Figure 2:**
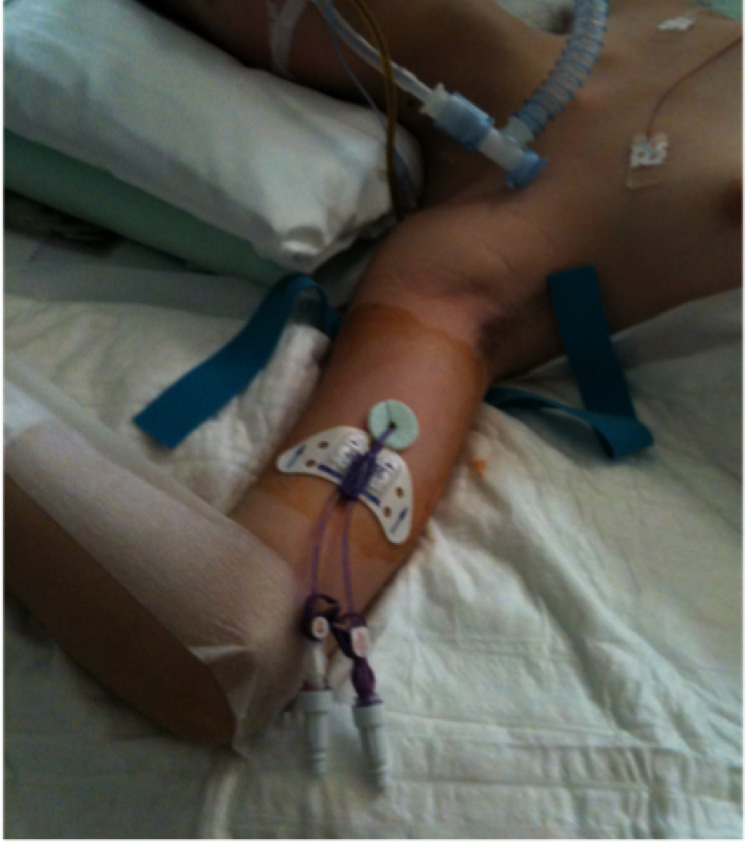
**Double-lumen power-injectable peripherally inserted central catheter inserted in a pediatric patient in the ICU**.

**Figure 3:**
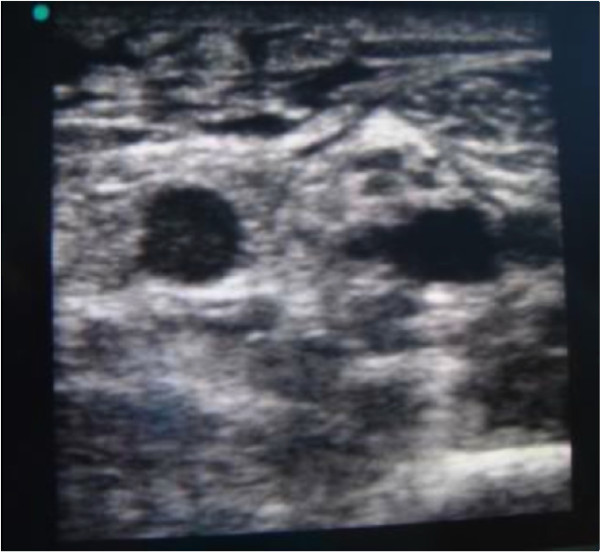
**Ultrasound scan at the mid-arm**. Left to right: basilic vein, brachial nerve, and brachial artery between two small brachial veins.

We considered both multiple-lumen and single-lumen catheters, of different sizes (from 4 to 6 Fr) and different brands, as long as they were power injectable and were inserted during the ICU stay (Figures 1 and 2). All PICCs were inserted according to the specific protocol defined by our hospital PICC team [9]. All of the catheters were inserted by ultrasound-guided puncture of the deep veins in the upper mid-arm (Figure 3), as recommended by current guidelines [3,10], using the micro-introducer technique. A standard 5 to 10 MHz linear ultrasound probe (adult patients) or a small 10 to 14 MHz hockey-stick ultrasound probe (children) was used. Veins with diameter = or > 4 mm were considered suitable for 4 Fr catheters, veins = or > 5 mm suitable for 5 Fr catheters and veins = or > 6 mm suitable for 6 Fr catheters. Maximal barrier precautions were consistently used during the procedure (cap, mask, sterile gown, sterile gloves, vast sterile field). The correct position of the tip of the catheter (that is, in proximity of the junction between the superior vena cava and the right atrium) was verified during the procedure using the intracavitary electrocardiography (EKG) method [11]; a post-procedural chest X-ray for checking the tip position was required only when the EKG method was not applicable (atrial fibrillation and/or no evident P-wave at the basal EKG). All PICCs were secured to the skin using sutureless devices, as currently recommended [12-14]. All procedures were performed by nurses or physicians specifically trained for PICC insertion. Maintenance of the line and dressing policies were carried out according to the intervention bundle for preventing line infections developed by the GAVeCeLT - Gruppo Accessi Venosi Centrali a Lungo Termine - and adopted by our hospital PICC team (preferential use of 2% chlorhexidine for antisepsis of the exit site, preferential use of transparent dressing, and so forth) [15]. Occlusion of the line was prevented by a specified policy of periodic flushing and locking with saline.

Acutely ill pediatric patients were also included in the study. Since 3 Fr power-injectable PICCs were not yet available in Italy at that time, power-injectable PICCs could be inserted in children only when an arm vein > 4 mm was available. In many acutely ill children, power-injectable PICCs had been inserted as direct central lines, by ultrasound-guided puncture of the internal jugular vein or of the brachio-cephalic vein, or as access to the inferior vena cava, by ultrasound-guided puncture of the femoral vein: all of these off-label uses of power-injectable PICCs were excluded from this analysis (only PICCs inserted into the upper mid-arm were considered).

According to our hospital policies, diagnosis of CRBSI was established by the differential time to positivity method (blood culture from the catheter becoming positive at least 120 minutes before the peripheral blood culture) or by direct culture of the tip of the catheter, should the catheter be removed or replaced over guidewire (culture of the same microorganism from the blood and from the tip of the catheter).

Diagnosis of catheter-related thrombosis was established by ultrasound examination (compression ultrasonography and duplex Doppler), performed only when clinically indicated by signs and symptoms suggesting venous occlusion (edema of the arm, PICC malfunction, unexplained local pain, and so forth). Catheter-related peripheral venous thrombosis was defined as the presence of occlusive thrombosis of the deep veins of the arm containing the PICC, extending or not to the axillary vein. Catheter-related central venous thrombosis was defined as the presence of any extent of thrombosis in the subclavian, internal jugular or brachio-cephalic vein or in the superior vena cava on the side of PICC insertion.

We collected all relevant data and information, including patient's age and disease, indication for PICC insertion, duration of the dwell time for the device, incidence of complications at insertion or during maintenance, and cause of removal. All data were included in a software-operated database and analyzed by standard descriptive statistics. Values are reported as the mean ± standard deviation.

According to the policy of our hospital, retrospective studies do not require approval from the Ethics Committee and do not require informed consent from the patient. Informed consent was obtained, however, from the patients represented in Figures 1 and 2 for publications of their images.

## Results

During the study period, 89 open-ended, power-injectable polyurethane PICCs were inserted in our ICUs. Sixty-five catheters were inserted in adult patients (50 triple-lumen 6 Fr, six double-lumen 5 Fr and nine single-lumen 4 Fr) and 24 catheters in pediatric patients (15 double-lumen 5 Fr, two double-lumen 4 Fr and seven single-lumen 4 Fr).

All PICCs were successfully inserted without major complications. Minor complications at insertion were local hematoma (three cases, 3.4%), repeated punctures of the vein (five cases, 5.6%), difficulty in progression of the catheter (four cases, 4.5%), and one malposition (one patient with no evident P-wave on basal EKG, where the intracavitary EKG method could not be used). This latter case required repositioning of the PICC by exchange over guidewire. A few adult patients had severe obesity (body mass index > 35, seven patients); in these patients PICCs were inserted without difficulty, mostly via the cephalic vein since brachial and basilica veins were too deep.

The average dwell time of the device in the ICU was 25 ± 12 days (median value 22 days): most catheters stayed in place for > 2 weeks and were removed when the patient was transferred to a non-intensive ward. No PICC was removed because of complications while in the ICU. One patient died while the PICC was still indwelling. In a few cases of adult patients, the PICC was left in place even after discharge from the ICU.

Most PICCs were used easily for high-flow intravenous infusions ( > 1,000 ml/hour, by infusion pump), as well as for measurement of the central venous pressure. In almost one-half of patients, PICCs were also used for injection of contrast medium during computed tomography scans. There were no relevant episodes of occlusion or of persistent difficulty in blood withdrawal; eight cases of partial obstruction were resolved by simple saline infusion under pressure. There was not a single case of CRBSI. Symptomatic catheter-related central venous thrombosis occurred only in one adult in the ICU (a patient with a hematological neoplastic disease, admitted to the ICU for respiratory failure); a second case occurred in a patient after his transfer to a non-intensive ward. Both episodes occurred within 10 days after PICC insertion. There were no dislodgements or accidental removals of the device while the patients were in the ICU; one case of accidental removal occurred after transfer of the patient to a non-intensive ward.

## Discussion

The first clinical experience with PICCs in the ICU was reported in 1996 [16]. At that time PICC insertion was usually performed at the antecubital fussa, without ultrasound guidance, but the results of this experience were particularly promising - especially in terms of minimal incidence of CRBSI (91 PICCs, 0.6 episodes per 1,000 catheter-days). The potential advantages of PICCs in the ICU have been described by other authors [7]; however, reports dealing specifically with the use of power-injectable PICCs [17,18], and particularly of power-injectable PICCs in the ICU [19], are scarced. To our knowledge, the present paper is the first clinical study reporting a detailed analysis of the complications associated with the use of power-injectable PICCs in adult and pediatric patients in the ICU.

Our rate of complications at insertion has been extremely low, especially considering the anatomical difficulties expected in acutely ill patients. Complications at insertion are known to be minimal when using both ultrasound guidance and the micro-introducer technique [2,10,20,21].

With regards to the incidence of CRBSI, evidence is accumulating that PICCs are associated with a lower rate of infection if compared with CVCs, probably because of an exit site that is less prone to contamination (upper mid-arm skin is characterized by a lower bacterial colonization if compared with skin at the neck or in the infra-clavicular area) [2,3]. This lower infection rate has also been shown in ICU patients. In a 2008 multicenter study including eight Spanish ICUs, the CRBSI rate of PICCs was significantly lower than that of CVCs (1.08 episodes vs. 3.83 episodes for 1,000 catheter-days), although ultrasound guided insertion was not used [22]. In a recent study carried out regarding 37 PICCs in a burn unit [23] the incidence of PICC-related bloodstream infection was 0 episodes, as compared with 6.6 episodes of CVC-related bloodstream infection for 1000 catheter-days. In another study in a surgical ICU [24], PICCs presented 2.2 episodes of CRBSI per 1,000 catheter-days versus 6 episodes in CVCs. Even in a recent clinical study [19], which reported a high incidence of PICC-related thrombosis (see below), there was not one episode of CRBSI in a cohort of 50 PICCs consecutively inserted into ICU patients. According to another recent paper in ICU patients [25], the occurrence of CRBSI appears to happen later in PICCs compared with CVCs (23 days vs. 13 days). As reported in similar papers on ICU patients [19,23], in our experience the rate of CRBSI was zero - although it must be stressed that our investigation was a noncontrolled retrospective study and did not aim to compare CVCs with PICCs in terms of infection rate.

Mechanical complications were also uncommon in our series. Dislodgement has been described as a problem only in studies not using ultrasound-guided insertion in the upper mid-arm and/or not using sutureless devices for PICC securement [16]. Also, mechanical damage such as ruptures usually occurs with silicon PICCs rather than with polyurethane PICCs [21]. The ultraresistant polyurethane of power-injectable PICCs appears to protect from mechanical damage, dislodgement and occlusion. In a recent clinical study [18], a decreased risk of occlusion and rupture was reported with power-injectable PICCs if compared with silicon and standard polyurethane PICCs.

A high incidence of clinically symptomatic PICC-related thrombosis has been recently reported by two papers, one dealing with non-power-injectable PICCs in the ICU [26] and the other with triple-lumen 6 Fr power-injectable PICCs in the ICU [19]. These findings are in contrast with the low incidence of symptomatic thrombosis reported by our present study and by a recent study of a series of 473 power-injectable PICCs [17]. This apparent contradiction may be explained by the observation that PICC-related venous thrombosis is known to be secondary to many different factors [2], including the use of ultrasound guidance during insertion, the choice of vein (the diameter of the vein should be at least three times the diameter of the PICC), the proper position of the tip, as well as the material of the catheter and the use of sutureless devices for securement. The low rate of symptomatic PICC-related thrombosis in our study may thus be explained by the consistent use of ultrasound guidance, by the careful choice of a vein of an appropriate diameter, by the intra-procedural verification of the position of the tip by the intracavitary EKG method, as well as by the use of a sutureless securement device in 100% of cases. On the contrary, in the papers quoted above, the same catheter size - either 5 Fr [26] or 6 Fr [19] - was adopted in every patient.

## Conclusions

This preliminary retrospective report shows that power-injectable PICCs can be successfully utilized in most ICU patients, even in those requiring high volumes of fluids, multiple intravenous lines and/or monitoring of the central venous pressure, as well as in children. Contraindications to PICC insertion are few, such as the need for an emergency central line, need for a central line with more than three lumens, and lack of availability of a vein > 4 mm in the upper mid-arm. CRBSI and catheter-related venous thrombosis appear to be uncommon. Most early and late complications can be successfully minimized by the adoption of an insertion protocol [27] including key recommendations such as ultrasound guidance, by the choice of a vein with appropriate caliber, by careful positioning the tip by the EKG method, and by the consistent use of sutureless securement devices.

Considering the results of this preliminary retrospective analysis, further prospective, controlled, randomized trials comparing the clinical outcome of CVCs with PICCs in intensive care patients are warranted.

## Key messages

• Power-injectable PICCs are a promising alternative option to standard CVCs in ICU patients.

• Power-injectable PICCs may have a role in many ICU patients, with few contraindications (emergency, need for more than three lumens, small deep veins of the arm, or local contraindications to brachial/basilic venipuncture).

• If inserted with a proper protocol (including ultrasound guidance, proper choice of vein, EKG guidance, aseptic technique, securement with sutureless device, and so forth), the insertion of PICCs is not associated with any relevant risk, even in patients with severe cardiorespiratory problems.

• If inserted with a proper protocol (see above), the risk of catheter-related infection is similar to or even lower than that with CVCs.

• If inserted with a proper protocol (see above), the risk of catheter-related thrombosis is similar to that with CVCs.

## Abbreviations

CRBSI: catheter-related bloodstream infection; CVC: central venous catheter; EKG: electrocardiography; PICC: peripherally inserted central catheter.

## Competing interests

The authors declare that they have no competing interests.

## Authors' contributions

The study was conceived, designed and coordinated by MPi and GS. The clinical data were collected by AB, MPo, DC and MPi. MPi, DGB, MGA and GS analyzed the data and prepared the manuscript. All authors have read and approved the manuscript for publication.
